# Association between antibiotic use, immune-related adverse events, and efficacy of immunotherapy in esophageal squamous cell carcinoma

**DOI:** 10.1007/s10147-026-03036-9

**Published:** 2026-04-27

**Authors:** Tomoaki Shirakawa, Hiroo Imai, Ken Saijo, Ryo Saito, Shiori Ishikawa, Iori Takahashi, Keigo Komine, Kota Ouchi, Yuki Kasahara, Sakura Taniguchi, Yuya Yoshida, Ryunosuke Numakura, Shonosuke Wakayama, Hidekazu Shirota, Masanobu Takahashi, Hisato Kawakami

**Affiliations:** 1 Department of Clinical Oncology, Tohoku University Graduate School of Medicine, Seiryo-machi 4-1, Aobaku, Sendai, 980-8575 Japan; 2 Department of Medical Oncology, Tohoku University Hospital, Seiryo-machi 4-1, Aobaku, Sendai, 980-8575 Japan

**Keywords:** Esophageal squamous cell carcinoma, Immune checkpoint inhibitor, Antibiotics, Immune-related adverse events

## Abstract

**Introduction:**

Immune checkpoint inhibitors (ICIs) are essential for treating esophageal squamous cell carcinoma (ESCC). As antibiotics (Abx) may reduce ICI efficacy, and immune-related adverse events (irAEs) relate to better outcomes, we investigated their association.

**Patients and methods:**

We retrospectively analyzed 121 advanced or metastatic ESCC patients treated with ICIs, assessing outcomes based on Abx use and irAEs.

**Results:**

Forty-one patients (33.9%) were in the Abx (+) group, showing a significantly shorter median progression-free survival (PFS) (2.5 vs. 6.5 months; *p* = 0.029) and lower disease control rate (36.8% vs. 60.9%; *p* = 0.0145) than those in the Abx (−) group. irAEs were less frequent in the Abx (+) group (29.3% vs. 58.8%; *p* = 0.0037). The positive impact of irAEs on ICI efficacy was significant in overall population [irAE(−) vs. irAE(+): PFS, 4.4 months (95% CI 2.5–5.3) vs. 8.8 months (95% CI 5.9–13.7); log-rank *p* = 0.001; Hazard ratio (HR) 1.76, 95% CI 1.14–2.74; *p* = 0.011; overall survival (OS), 10.6 months (95% CI 7.0–13.1) vs. 18.1 months (95% CI 9.2–25.9); log-rank* p* = 0.043; HR 1.61, 95% CI 1.01–2.55; *p* = 0.046] but diminished if received Abx. Notably, the Abx (−)/irAE (+) group had the best outcomes, while the Abx (+)/irAE (−) group had the worst PFS (8.7 vs. 2.4 months; HR 2.07; *p* = 0.011) and OS (18.1 and 6.8 months; HR 1.89; *p* = 0.036).

**Conclusion:**

Antibiotic use was linked to reduced ICI efficacy and fewer irAEs, suggesting impaired immune activation in ESCC patients.

**Supplementary Information:**

The online version contains supplementary material available at 10.1007/s10147-026-03036-9.

## Introduction

Esophageal squamous cell carcinoma (ESCC) is the seventh leading cause of cancer-related deaths worldwide [1], and the prognosis for patients with metastatic or recurrent disease remains poor. The advent of immune checkpoint inhibitors (ICIs) has significantly improved outcomes in this setting [2]. For patients with unresectable, advanced, metastatic, or recurrent ESCC (mESCC), the current standard first-line treatment includes 5-fluorouracil and cisplatin, combined with pembrolizumab or nivolumab, both anti-PD-1 antibodies [3]. In addition, the chemotherapy-free combination of nivolumab and ipilimumab, an anti-CTLA-4 antibody, has emerged as another standard regimen [3], suggesting that mESCC is a highly immunogenic tumor. Furthermore, in patients with locally advanced ESCC who receive neoadjuvant chemoradiotherapy followed by curative surgery, adjuvant nivolumab monotherapy is recommended for those who do not achieve a pathological complete response [4]. Collectively, these developments underscore the pivotal role of ICIs in ESCC treatment, both in locally advanced and metastatic settings, and highlight the need to optimize their therapeutic efficacy to improve patient outcomes further.

Several factors have been proposed to influence ICI efficacy, mostly tumor-intrinsic factors such as tumor mutational burden (TMB) and combined positive score (CPS) [5, 6]. Other factors include host-related systemic immune markers such as the neutrophil-to-lymphocyte ratio, serum albumin, and C-reactive protein levels [7, 8], which draw attention as potential indicators of immune competence or exhaustion. A more direct surrogate for intact immune activation is the occurrence of immune-related adverse events (irAEs) that are unique to ICI. Several observational studies have demonstrated a positive association between the development of irAEs and improved clinical outcomes with ICI treatment [9–12]. In contrast, antibiotic use has been implicated as a negative host-related factor, with multiple studies reporting an association between antibiotic exposure and diminished ICI efficacy [13–17], potentially due to the disruption of the gut microbiota. However, the underlying mechanisms remain poorly understood. In clinical practice, patients with mESCC frequently receive antibiotics, particularly for aspiration pneumonia; however, robust evidence regarding the true impact of antibiotics on ICI effectiveness in mESCC remains limited. Moreover, the relationship between antibiotic use and the development of irAEs, which are clinical events associated with ICI efficacy, remains unclear.

To address these gaps, we conducted a retrospective study to evaluate the impact of antibiotic use and irAE occurrence on the efficacy of ICIs in patients with mESCC after adjusting for relevant clinical background factors. Additionally, we explored the potential interaction between antibiotic use and irAE development, and how this relationship may influence treatment outcomes in this population.

## Materials and methods

This retrospective study was conducted in the Department of Medical Oncology, Tohoku University Hospital, Japan. Medical records from 2020 to 2024 were reviewed to evaluate the efficacy and safety of ICIs in patients with mESCC. This study was approved by the Ethics Committee of Tohoku University Hospital (approval number: 2023–1-169).

### Inclusion criteria

Patients were eligible for inclusion if they met the following criteria: histologically confirmed diagnosis of mESCC, received at least one cycle of an ICI-containing regimen, and underwent at least one imaging evaluation to assess treatment response. The antibiotic-treated group included patients who received antibiotics within one month before or after the initiation of ICI therapy. The antibiotic-untreated group included patients who did not receive antibiotics within the predefined peri-treatment window. This peri-treatment window was selected a priori to capture antibiotic use most likely to influence the gut microbiota and host immune status at the time of ICI initiation, which is considered a critical period for priming antitumor immune responses.

Patients who received antibiotics more than 1 month after ICI initiation were classified as antibiotic-untreated for the purpose of this analysis. This approach was adopted to minimize time-dependent bias and immortal time bias, as antibiotics administered later during treatment are often prescribed for intercurrent infections occurring after treatment response or disease progression and are unlikely to reflect baseline immune competence at the start of ICI therapy.

### Treatment regimens

The ICI regimens used in this study included the following: nivolumab (NIVO) monotherapy (administered intravenously at a dose of 240 mg/body every 2 weeks or 480 mg/body every 4 weeks); nivolumab plus ipilimumab (IPI) combination therapy (NIVO at 360 mg/body plus IPI at 1 mg/kg IV on day 1, followed by NIVO alone at 360 mg/body on day 22); NIVO plus FP regimen [NIVO 480 mg/body and cisplatin 80 mg/m^2^ on day 1, followed by a continuous IV infusion of 5-fluorouracil (5-FU) at 800 mg/m^2^ daily for 4 days which was repeated every 4 weeks]; pembrolizumab plus FP regimen (pembrolizumab 200 mg/body and cisplatin 80 mg/m^2^ on day 1, followed by 5-FU 800 mg/m^2^/day for 4 days repeated every 3 weeks for up to 6 cycles. Subsequently, pembrolizumab was continued along with 5-FU alone.

### Outcome measures

Treatment response was assessed using the Response Evaluation Criteria in Solid Tumors (RECIST), version 1.1. PFS was defined as the time from the initiation of ICIs to disease progression or death from any cause. Overall survival (OS) was defined as the time from the initiation of ICI therapy to death from any cause.

### Statistical analysis

PFS and OS were estimated using the Kaplan–Meier method. A stratified two-sided log-rank test was used to compare the treatment groups, and hazard ratios (HRs) were calculated using the Cox proportional hazards regression model. The overall response rate (ORR) and disease control rate (DCR) were compared between the two groups using Fisher’s exact test. irAEs were defined as treatment-related events with a likely immune origin that required intervention (e.g., steroid therapy) and were graded according to the Common Terminology Criteria for Adverse Events (CTCAE), version 4.0.

Differences in patient age were assessed using Student's *t*-test. Univariate and multivariate analyses were performed to identify the factors associated with clinical outcomes. All statistical analyses were conducted using JMP^®^ 11 software (SAS Institute Inc., Cary, NC, USA). A two-sided p-value of < 0.05 was considered statistically significant.

## Results

### Patient characteristics

A total of 121 patients with mESCC were included in this study. Patient characteristics are summarized in Table 1. ICIs were administered as first-line therapy in 41 (33.9%) patients, as second-line therapy in 70 (57.9%) patients, and as third- or later-line therapy in 10 (8.3%) patients. Ninety-four (77.7%) patients received ICI therapy, whereas 27 (22.3%) received ICIs in combination with chemotherapy. Among them, 41 (33.9%) had a history of antibiotic treatment (Abx [+] group), whereas 80 (66.1%) did not (Abx [+] group). In both groups, over 80% of the patients were male, and more than 90% had an Eastern Cooperative Oncology Group performance status (ECOG PS) of 0 or 1. The distribution of treatment lines was comparable between the Abx (+) and Abx (−) groups. Antibiotic use was not associated with the ICI treatment line or use of other concomitant medications. There were no significant differences between the groups in terms of the proportion of patients with a neutrophil-to-lymphocyte ratio (NLR) < 4, the presence of ≥ 2 metastatic organs, a tumor proportion score (TPS) ≤ 1, or in the rate of antacid use.

**Table 1:** Patient characteristics

	Abx (+)	Abx (−)	*p*-value	SMD
	n = 41 (%)	n = 80 (%)		
Sex				
Male	38 (92.7)	66 (82.5)	0.1271	0.31
Female	3 (7.3)	14 (17.5)		
Age (range)	69.0 (46–85)	67.9 (48–87)	0.6576	0.05
PS				
0 or1	39 (91.2)	76 (95.0)	0.3314	0.15
> 2	4 (9.8)	4 (5.0)		
ICI regimen				
Nivolumab	22 (53.7)	48 (60.0)	0.5037	0.12
Nivolumab plus ipilimumab	9 (22.0)	15 (18.8)	0.676	0.08
ICI plus FP	10 (24.4)	17 (21.3)	0.6946	0.07
Treatment line				
First	14 (34.1)	27 (33.8)	0.9652	0.01
Second	23 (56.1)	47 (58.8)	0.7797	0.05
> Third	4 (9.8)	6 (7.5)	0.6697	0.08
NLR				
≤ 4	19 (46.3)	47 (58.8)	0.1945	0.25
> 4	22 (53.7)	33 (41.3)		
Number of organs with metastases				
≤ 1	19 (46.3)	44 (55.0)	0.3669	0.17
≥ 2	22 (53.7)	36 (45.0)		
TPS				
1 ≤	5 (12.2)	13 (16.3)	0.553	0.12
1 >	8 (19.5)	15 (18.8)	0.9194	0.02
Unknown	28 (68.3)	52 (65.0)	0.7172	0.07
Antacids				
Yes	40 (97.6)	72 (90.0)	0.1335	0.32
No	1 (2.4)	8 (10.0)		
Type of antibiotics				
Beta-lactamase inhibitor	14 (34.1)			
Cephem	12 (29.3)			
Quinolone	6 (14.6)			
Penicillin	5 (12.2)			
Carbapenem	3 (7.3)			
ST	1 (2.4)			
Reason for antibiotics using				
Infection	21 (51.2)			
Prophylactic using	20 (48.8)			

*SMD* standardized mean difference, *ECOG PS* European clinical oncology group performance status, *ICI* immune checkpoint inhibitor, *FP 5FU* plus cisplatin combination regimen, *NLR* neutrophil–lymphocyte ratio, *TPS* tumor proportion score, *ST* trimethoprim-sulfamethoxazole

### Treatment efficacies of ICIs

In the ITT population (*N* = 121), the median PFS was 5.3 months (95% CI 4.2–6.6). The median PFS was 5.9 months (95% CI 4.9–11.0) in patients who received ICIs as first-line treatment (*n* = 41), 5.3 months (95% CI 4.1–6.3) in those who treated as the second line (*n* = 70), and 4.6 months (95% CI 1.9–2.8) in those who treated as the third-line or later setting. There was no significant difference in PFS between the different ICI treatment groups. In the ITT population (*N* = 121), the median OS was 11.0 months (95% CI 8.8–14.6). The median OS was 25.9 months (95% CI 10.7–30.1) in patients who received ICIs as first-line treatment, 10.1 months (95% CI 8.4–13.6) in those who treated as the second line, and 8.6 months (95% CI 4.0–20.6) in those who treated as the third-line or later setting (Supplementary Fig. 1a and 1b). Among the 107 patients with measurable lesions evaluated using the RECIST criteria, the ORR and DCR were 25.2% and 52.3%, respectively.

### Association between antibiotic use and the therapeutic efficacy of ICIs

**Fig. 1:**
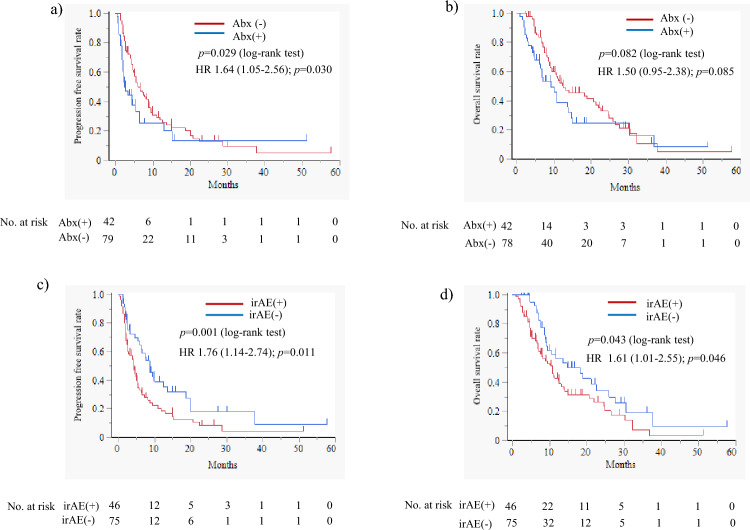
Kaplan–Meier curves showing **a** PFS and **b** OS for the antibiotics-treated group and **c** PFS and **d** OS for the irAE-positive and irAE-negative groups

First, we evaluated the effect of antibiotic use on the efficacy of ICI. The median PFS was significantly shorter in the Abx (+) group compared with the Abx (−) group [2.5 months (95% CI 1.8–5.3) vs. 6.5 months (95% CI 4.7–8.8); log-rank *p* = 0.029; HR 1.64 (95% CI 1.05–2.56); *p* = 0.030; Fig. 1a]. The median OS in the Abx (+) vs. the Abx (−) group showed a trend towards to a shorter OS [9.2 months (95% CI 6.2–13.7) vs.12.5 months (95% CI 9.3–21.2)], but it was not statistically significant [log-rank *p* = 0. 085; HR 1.50 (95% CI 0.95–2.38); *p* = 0.085; Fig. 1b]. The Abx (+) group consistently showed poorer outcomes than the Abx (−) group, with a numerically lower ORR (18.4% vs. 30.4%; *p* = 0.1898) and significantly lower DCR (36.8% vs. 60.9%; *p* = 0.0145; Table 2).

**Table 2:** ORR and DCR according to the presence or absence of antibiotic use and irAE incidence

Best response	Abx (+) n = 38	Abx (−) n = 69	*p*-value	irAE (−) n = 65	irAE (+) n = 42	*p*-value
CR	0	1		0	1	
PR	7	20		18	14	
SD	7	22		10	15	
PD	24	26		37	12	
ORR	18.4	30.4	0.1898	27.7	35.7	0.2134
DCR	36.8	60.9	0.0145	43.1	71.4	0.0281

The *p-*values were determined with Fisher’s exact test

*Abx* antibiotics, *irAE* immune related adverse event, *CR* complete response, *PR* partial response, *SD* stable disease, *PD* progressive disease, *ORR* objective response rate, *DCR* disease control rate

### Association between the occurrence of irAE and the therapeutic efficacy of ICIs

Next, we examined the association between the incidence of irAEs and the therapeutic response to ICIs. No correlation was observed between the occurrence of irAEs and the line of ICI treatment (Supplementary Table 1). The irAE (−) group persistently exhibited inferior outcomes compared with the irAE (+) group, with a numerically lower ORR (27.7% vs. 35.7%; *p* = 0.2134) and a significantly lower DCR (43.1% vs. 71.4%; *p* = 0.0281; Table 2). PFS was significantly shorter in the irAE (−) vs. the irAE (+) group [median 4.4 months (95% CI 2.5–5.3) vs. 8.8 months (95% CI 5.9–13.7); log-rank *p* = 0.001; HR 1.76, 95% CI 1.14–2.74; *p* = 0.011; Fig. 1c]. Similarly, overall survival was significantly shorter in the irAE (−) vs. the irAE (+) group [median 10.6 months (95% CI 7.0–13.1) vs. 18.1 months (95% CI 9.2–25.9); log-rank *p* = 0.043; HR 1.61, 95% CI 1.01–2.55; *p* = 0.046; Fig. 1d].

### Inverse association between antibiotics use and irAE occurrence

We examined the association between antibiotic use and the occurrence of irAEs. We found that the overall incidence of irAEs was significantly lower in the Abx (+) than in the Abx (−) group (29.3% vs. 58.8%, *p* = 0.0037), suggesting that antibiotic use was inversely associated with irAE incidence (Table 3). Among the various types of irAEs observed, the incidence of hypothyroidism was significantly lower in the Abx (+) than in the Abx (−) group (2.4% vs, 15.4%, *p* = 0.0173). The time from the initiation of ICI therapy to the onset of irAEs did not differ between the two groups (log-rank, *p* = 0.831; Supplementary Fig. 2).

**Table 3:** Incidence of irAEs with or without antibiotics

irAE	Abx (+) n = 41		Abx (−) n = 80		*p*-value
	Grade		Grade		
	Any (%)	> 3 (%)	Any (%)	> 3 (%)	
Total (%)	12 (29.3)	7 (17.1)	47 (58.8)	13 (16.3)	0.0037
Interstitial pneumonia	7 (17.1)	5 (12.2)	6 (7.5)	3 (3.8)	0.1075
Myositis	2 (4.9)	1 (2.4)	2 (2.5)	0 (0.0)	0.4886
Hypothyroidism	1 (2.4)	0 (0.0)	14 (15.4)	0 (0.0)	0.0173
Dermatitis	1 (2.4)	1 (2.4)	9 (11.3)	5 (6.3)	0.0957
Arthritis	1 (2.4)	0 (0.0)	1 (1.3)	0 (0.0)	0.6273
Hepatitis	0 (0.0)	0 (0.0)	3 (3.8)	2 (2.5)	0.2093
Colitis	0 (0.0)	0 (0.0)	3 (3.8)	1 (1.3)	0.2093
Hypopituitarism	0 (0.0)	0 (0.0)	4 (5.0)	0 (0.0)	0.1454
Hypoadrenalism	0 (0.0)	0 (0.0)	2 (2.5)	0 (0.0)	0.3073
Type 1 diabetes mellitus	0 (0.0)	0 (0.0)	1 (1.3)	1 (1.3)	0.4722
Pancreatitis	0 (0.0)	0 (0.0)	1 (1.3)	1 (1.3)	0.4722

*Abx* antibiotics, *irAE* immune related adverse events

### Multivariate analyses for PFS and OS

Next, we performed a multivariate analysis, including clinically important baseline characteristics, antibiotic use, and the occurrence of irAEs as covariates. In multivariable Cox proportional hazards analyses adjusting for clinically relevant covariates, antibiotic use and the absence of irAEs remained independently associated with shorter PFS, while the absence of irAEs was independently associated with shorter OS (Table 4). We found that PFS was significantly shorter in patients treated with NIVO or NIVO + IPI than in those treated with ICIs + FP (*p* = 0.006 and *p* = 0.027), in patients who received antibiotics than in those who did not (*p* = 0.006 and *p* = 0.009), and in patients without irAEs than in those with irAEs (*p* = 0.019 and *p* = 0.003). In contrast, our analysis demonstrated that OS was significantly shorter in patients treated with the NIVO + IPI regimen than in those treated with ICIs + FP (*p* = 0.008) and in patients without irAEs (*p* = 0.014). These findings indicate that antibiotic use and the occurrence of irAEs are independent prognostic factors for PFS and for OS, respectively.

**Table 4:** Univariate and multivariate analyses for PFS or OS

Variable	PFS					OS			
	Univariate		Multivariate			Univariate		Multivariate	
	HR (95%CI)	*p*-value	HR (95%CI)	*p*-value		HR (95%CI)	*p*-value	HR (95%CI)	*p*-value
Sex									
Male	0.950	0.865	0.989	0.972		0.820	0.602	0.825	0.585
Female	1.050		1.011			1.220		1.212	
Regimen									
ICIs	2.323	0.006	2.833	0.027		4.971	0.002	3.918	0.008
ICI plus FP	0.430		0.353			0.201		0.255	
ECOG PS									
0 or 1	0.853	0.799	0.788	0.625		0.523	0.171	0.579	0.299
≥ 2	1.172		1.270			1.913		1.727	
Treatment line									
1st	0.607	0.031	0.781	0.372		0.690	0.140	0.723	0.419
≥ 2nd	1.645		1.019			1.450		1.383	
Number of metastatic organs									
≤ 1	0.849	0.446	0.875	0.558		0.905	0.696	0.870	0.582
≥ 2	1.178		1.142			1.105		1.150	
Antibiotic use									
Yes	1.856	0.006	1.825	0.009		1.680	0.046	1.344	0.296
No	0.539		0.461			0.595		0.744	
irAE incidence									
Yes	0.587	0.019	0.488	0.003		0.677	0.134	0.531	0.014
No	1.706		2.049			1.478		1.884	
NLR									
< 4	0.719	0.127	0.778	0.262		0.680	0.124	0.859	0.524
≥ 4	1.390		1.286			1.471		1.166	
Antacid use									
Yes	1.148	0.711	1.227	0.640		1.498	0.351	1.943	0.170
No	0.871		0.815			0.668		0.515	

The *p-*values were determined with Fisher’s exact test

*PFS* progression free survival, *OS* overall survival, *HR* hazard ratio, *ECOG PS* European clinical oncology group performance status, *irAE* immune related adverse events, *NLR* neutrophil–lymphocyte ratio

**Fig. 2:**
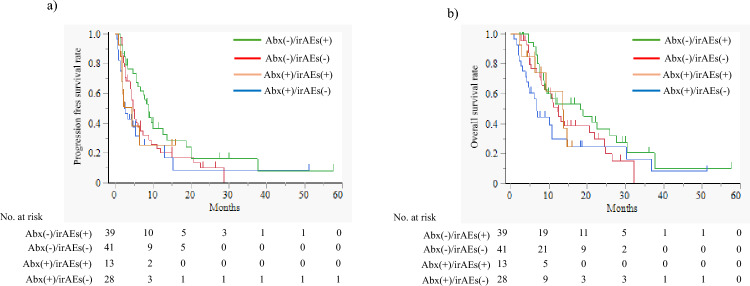
Kaplan–Meier curves showing **a** PFS and **b** OS for the following four groups: antibiotics(−)/irAE(+), antibiotics(−)/irAE(−), antibiotics(+)/irAE(+) and antibiotics (+)/irAE(−)

Therefore, we divided our mESCC cohort into four groups according to antibiotic use [(+) or (−)] and the occurrence of irAEs [(+) or (−)], and examined the impact of these factors on ICI efficacy. We found that the Abx (−)/irAEs (+) group demonstrated the best PFS [8.7 months (95% CI 5.5–12.6); Fig. 2a] whereas the Abx (+)/irAEs (−) group showed the worst PFS [2.4 months (95% CI 1.8–6.4); Fig. 2a]. For OS, a significant difference was again observed between the Abx (−)/irAEs (+) group and the Abx (+)/irAEs (−) group [18.1 months (95% CI 8.7–25.9) vs. 6.8 months (95% CI 4.0–10.6); log-rank *p* = 0.036; HR 1.89, 95% CI 1.04–3.41; *p* = 0.034; Fig. 2b). Although there were no significant differences in the ORR among the four groups, the DCR in the Abx (−)/irAE (+) group was significantly higher than that in the Abx (−)/irAE (−) and Abx (+)/irAE (−) groups (*p* = 0.0073 and *p* = 0.0085, respectively; Supplementary Table 2). In contrast, there were no differences in PFS, OS, ORR, or DCR between the Abx (+) irAE (+) and Abx (−) irAE (−) groups (Fig. 2a and b). Consistent results were obtained after adjusting for potential confounding factors, including C-reactive protein (CRP) level, neutrophil-to-lymphocyte ratio (NLR), age, and Eastern Cooperative Oncology Group performance status (ECOG PS), using propensity score matching (Supplementary Fig. 3, Supplementary Tables 3, 4 and 5).

## Discussion

While the negative impacts of antibiotic use on the efficacy of ICIs have been reported in various cancer types, data on mESCC have been limited to small retrospective cohorts (*N* = 49) [18]. In this study involving a substantial cohort of patients with mESCC (*N* = 121), we found that antibiotic use was associated with a significantly worse DCR, PFS, and OS. In our cohort, approximately one-third of the patients received antibiotics with 51% administered for the treatment of infection and 49% as prophylaxis for suspected infections. While there were no significant differences in PS between the Abx (+) and Abx (−) groups, unmeasured confounders related to baseline condition could not be excluded, which may partly explain the poor OS observed in the Abx (+) group. However, a particularly interesting finding was that the Abx (+) group had significantly worse PFS and tumor control (i.e., DCR; ORR was numerically lower but not statistically significant), suggesting that antibiotic use may not only be a prognostic factor, but also a potential predictive factor for ICI efficacy. One possible mechanism is that antibiotics disrupt the gut microbiota, leading to dysbiosis, which in turn impairs the systemic immune response to ICIs. Several studies have indicated a close link between the gut microbiota composition and ICI efficacy [19–21], warranting further investigation into the immunological consequences of antibiotic-induced dysbiosis.

Similarly, the association between irAEs and favorable ICI outcomes has been well documented in other malignancies; however, reports on mESCC remain limited. Consistent with prior findings [9–12, 22], our study showed that patients who experienced irAEs had significantly better PFS, OS, and DCR (although not ORR). Although improved survival in patients with irAEs has been previously reported [9–12, 22], data on DCR are scarce. Our results suggest that irAEs may serve not only as prognostic indicators but also as potential on-treatment biomarkers for ICI efficacy. irAEs reflect the host systemic immune activation triggered by ICIs, suggesting that the host immune system is responsive to treatment.

From a methodological perspective, it should be noted that irAEs are post-baseline events. In the present study, irAE occurrence was therefore treated as a binary covariate, which may introduce potential guarantee-time or immortal time bias. However, the primary objective of this study was to evaluate the clinical association between irAE development and ICI efficacy in routine clinical practice, rather than to establish a strict causal relationship. Importantly, the time from ICI initiation to irAE onset did not differ significantly between the antibiotic-treated and untreated groups (Supplementary Fig. 2), suggesting that differential timing of irAE occurrence was unlikely to account for the observed differences in clinical outcomes. These findings should therefore be interpreted as hypothesis-generating, and future studies using time-dependent covariate models or landmark analyses in larger cohorts will be required to more rigorously address the temporal relationship between irAE development and treatment outcomes.

Regarding the relationship between antibiotic use and irAE incidence, a large-scale study (*N* = 767) reported that antibiotic exposure increased the risk of irAEs [23]. However, this study was conducted mainly in patients with non-small cell lung cancer (*n* = 340), in whom interstitial pneumonitis is a common irAE. In clinical practice, antibiotics are often administered empirically when suspicious pulmonary shadows appear during ICI treatment, and the diagnosis of irAEs is based on the response to treatment [24]. This report includes numerous cases in which antibiotics were administered after the onset of irAEs; thus, antibiotic use was associated with the occurrence of irAEs. In our analysis, antibiotic use was significantly associated with a reduced incidence of irAEs. Differences in the timing of antibiotic exposure may have accounted for this discrepancy. Specifically, our study defined Abx exposure as occurring within 1 month before or after ICI initiation, whereas that study used a window of 3 months, possibly including antibiotics administered after the onset of irAEs. Furthermore, mESCC data in that study were extremely limited (*n* = 63), and a positive correlation between antibiotic use and irAE incidence was not clearly reported (odds ratio, 0.98). Future studies examining the association between antibiotics and irAEs should carefully consider the indications and timing of antibiotic use.

Notably, patients who received antibiotics but did not develop irAEs had particularly poor outcomes in terms of ICI efficacy and survival. These findings were replicated in a propensity score–matched analysis adjusted for baseline characteristics. We observed no difference in time to irAE onset between the Abx (+) and Abx (−) groups. It is conceivable that antibiotic-induced dysbiosis attenuates immune activation by ICIs, resulting in suboptimal antitumor effects. This has major clinical implications for mESCC, a disease for which ICIs are central to the treatment of locally advanced or metastatic cases. Given that the therapeutic effect of ICIs largely determines prognosis, unnecessary or prophylactic antibiotic use should be avoided. Even when antibiotics are deemed necessary, clinicians should be aware of the potential for dysbiosis and consider countermeasures, such as the co-administration of resistant probiotics. We also found that the treatment outcomes of the Abx (+) irAE (+) group were similar to those of the Abx (−) irAE (−) group, suggesting that irAEs may predict ICI effectiveness even in the presence of antibiotic use. Therefore, although antibiotics may reduce the occurrence of irAEs, this is not beneficial, given the associated loss of ICI efficacy. However, further research is needed to identify biomarkers that reflect host immune competence and to inform the appropriate use of antibiotics during ICI therapy.

This study had several limitations. First, it was a retrospective analysis, which is subject to various biases, including the lack of standardization in antibiotic use and the use of antacids. Antacid use has also been reported to be a negative factor in ICI efficacy [25, 26]. Second, there was heterogeneity in the ICI-based treatment regimens and in the treatment lines at which ICIs were administered, including ICI monotherapy, nivolumab plus ipilimumab, and ICI combined with FP chemotherapy. These regimens differ in their biological mechanisms, toxicity profiles, and clinical indications, and treatment selection may reflect differences in patient condition that are not fully captured by baseline variables. This heterogeneity may have introduced residual confounding in OS analyses and could partly explain why our hypothesis that the longest OS would be observed in the Abx (−)/irAE (+) group was not clearly demonstrated. To mitigate this issue, treatment regimen was explicitly included as a covariate in multivariable Cox proportional hazards models and in propensity score matching. Notably, antibiotic use and irAE occurrence remained independently associated with PFS and/or OS after adjustment for treatment regimen, and the direction of these associations was consistent across analyses. Furthermore, when treatment outcomes were analyzed separately according to ICI treatment line (first-line vs. second-line or later), the results were consistent with the overall findings (data not shown). Nevertheless, residual confounding related to treatment heterogeneity cannot be completely excluded, and our findings should be interpreted as hypothesis-generating rather than definitive evidence of causality.

## Conclusion

In patients with mESCC treated with ICIs, antibiotic use was associated with reduced efficacy and a lower incidence of irAEs, possibly because of impaired immune activation. Notably, irAE development predicted favorable outcomes regardless of antibiotic exposure. As antibiotic use is often unavoidable in patients with mESCC, there is a pressing need to develop biomarkers that can monitor host immune competence and guide appropriate antibiotic use before or during ICI therapy.

## Conflict of interest

Hisato Kawakami: *Consulting or advisory fees*: Bristol-Myers Squibb Co. Ltd.; Bayer Yakuhin Ltd.; Eli Lilly Japan K.K.; MSD K.K.; Ono Pharmaceutical Co. Ltd.; Chugai Pharmaceutical Co. Ltd.; Daiichi Sankyo Co. Ltd.; Merck Biopharma Co., Ltd.; Takeda Pharmaceutical Co. Ltd.; Takata Pharmaceutical; Taiho Pharmaceutical Co. Ltd.; Otsuka Pharmaceutical Co., Ltd.; Nippon Kayaku Co. Ltd.; GlaxoSmithKline K.K.; Amgen; Novartis International AG; Astellas Pharma Inc.; BeOne Medicine Japan; AstraZeneca K.K.; Miyarisan Pharmaceutical. Co. Ltd. *Research funding*: Eisai Co. Ltd.; Bristol-Myers Squibb Co. Ltd.; Kobayashi Pharmaceutical. Co., Ltd.; Astellas Pharma Inc.; Hitachi, Ltd. Ono Pharmaceutical Co. Ltd.; Boehringer Ingelheim Japan.Masanobu Takahashi: *Research funding*: Ono Pharmaceutical, MSD, and Boehringer Ingelheim. Ken Saijo: Honoraria: Novartis Pharma. The remaining authors declare no conflict of interest.

## Supplementary Information

Below is the link to the electronic supplementary material.

Supplementary file1 (DOCX 58 KB)

Supplementary file2 Supplementary Fig. 1. Kaplan–Meier curves showing: a) PFS in all cases, b) PFS stratified by ICI treatment line, c) OS in all cases, and d) OS stratified by ICI treatment line. Supplementary Fig. 2. Cumulative incidence rate of irAEs in the antibiotics-treated and the antibiotics-untreated groups. This figure presents a time-to-event analysis evaluating the time to the first irAE occurrence on a per-patient basis. Accordingly, the number at risk differs from the total number of irAE events reported in Table 3, where all irAEs, including multiple events occurring in the same patient, were counted. Supplementary Fig. 3. Kaplan-Meier curves showing a) PFS and b) OS for the antibiotics-treated group and c) PFS and d) OS for the irAE-positive and irAE-negative groups after propensity score matching. (PPTX 153 KB)

## Data Availability

The raw data supporting the conclusions of this article will be made available by the first author (tomoaki.shirakawa.c2@tohoku.ac.jp) and corresponding author (hiroo.imai.d8@tohoku.ac.jp) without undue reservation.
