# Supplementary material for: Association between antibiotic use, immune-related adverse events, and efficacy of immunotherapy in esophageal squamous cell carcinoma
Source: Int J Clin Oncol. 2026 Apr 27;31(7):1258–66. doi: 10.1007/s10147-026-03036-9 (PMC13303444; doi:10.1007/s10147-026-03036-9)
Supplement: Supplementary file 1 — Supplementary file1 (DOCX 58 KB) [file 10147_2026_3036_MOESM1_ESM.docx]

Supplementary Table 1. Incidence rate of irAEs by ICI treatment line

| Treatment line | irAE-negative (%) | irAE-positive (%) | *p*-value |
| --- | --- | --- | --- |
| 1st | 29 (38.6) | 12 (26.1) | 0.1558 |
| 2nd | 39 (52.0) | 31 (67.4) | 0.4978 |
| > 3rd | 7 (9.3) | 3 (6.5) | 0.5856 |
| irAE, immune related adverse event, The *p-*values were determined with Fisher’s exact test. | | | |

Supplementary Table 2. ORR and DCR according to the presence or absence of antibiotics and irAEs.

|  | Abx (-)/irAE (+) | Abx (-)/irAE (-) | Abx (+)/irAE (+) | Abx (+)/irAE (-) |
| --- | --- | --- | --- | --- |
| CR | 1 | 0 | 0 | 0 |
| PR | 8 | 12 | 3 | 5 |
| SD | 15 | 12 | 1 | 6 |
| PD | 9 | 17 | 9 | 17 |
| ORR (%) | 27.3 | 29.3 | 23.1 | 17.9 |
| DCR (%) | 72.7 | 58.5 | 30.8 | 39.3 |
| Abx, antibiotics; irAE, immune related adverse event; CR, complete response; PR, partial response; SD, stable disease; PD, progressive disease; ORR, objective response rate; DCR, disease control rate | | | | |

Supplementary Table 3. PFS and OS according to the presence or absence of antibiotics and irAEs.

| Abx | irAEs | PFS  (95% CI) | *p*-value  (log-rank) | Hazard ratio  (95%CI; *p*-value) | OS  (95%CI) | *p*-value  (log-rank) | Hazard ratio  (95%CI; *p*-value) |
| --- | --- | --- | --- | --- | --- | --- | --- |
| absence | presence | 8.7 months  (5.5-12.6) | Reference | Reference | 18.1 months  (8.7–25.9) | Reference | Reference |
| absence | absence | 4.8 months  (3.8–6.6) | 0.039 | 1.47  (1.11-2.43; *p*=0.032) | 12.3 months  (8.1–21.8) | 0.197 | 1.38  (0.80-2.39; *p*=0.243) |
| presence | presence | 4.4 months  (1.7–NA) | 0.049 | 1.61  (1.07-4.03; *p*=0.042) | 13.6 months  (6.3–NA) | 0.244 | 1.48  (0.63-3.48; *p*=0.034) |
| presence | absence | 2.4 months  (1.8–6.4) | 0.011 | 2.07  (1.16-3.67; *p*=0.042]) | 6.8 months  (4.0–10.6) | 0.036 | 1.89  (1.04-3.41; *p*=0.034) |
| Abx, antibiotics; irAEs, immune related adverse events; PFS, progression free survival; OS, overall survival | | | | | | | |

Supplementary Table 4. Patient characteristics in propensity score matching

|  | Abx (+) | Abx (-) | *p*-value | SMD |
| --- | --- | --- | --- | --- |
|  | n = 31 | n = 31 |  |  |
| Sex |  |  |  |  |
| Male | 28 (90.3) | 24 (77.4) | 0.1672 | 0.35 |
| Female | 3 (9.7) | 7 (22.6) |  |  |
| Age (range) | 69.5 (46-85) | 67.8 (47-83) | 0.4657 |  |
| ECOGPS |  |  |  |  |
| 0 or1 | 31 (100.0) | 30 (96.8) | 0.3134 | 0.25 |
| >2 | 0 (0.0) | 1 (3.2) |  |  |
| ICI regimen |  |  |  |  |
| Nivolumab | 14 (45.2) | 19 (61.3) | 0.2031 | 0.33 |
| Nivolumab plus ipilimumab | 7 (22.6) | 5 (16.1) | 0.5203 | 0.16 |
| ICI plus FP | 10 (32.3) | 7 (22.6) | 0.3931 | 0.22 |
| Treatment line |  |  |  |  |
| First | 13 (41.9) | 10 (32.3) | 0.4303 | 0.20 |
| Second | 14 (45.2) | 18 (58.1) | 0.3094 | 0.26 |
| >Third | 4 (12.9) | 3 (9.7) | 0.6882 | 0.01 |
| NLR |  |  |  |  |
| <4 | 18 (58.1) | 18 (58.1) | 1.0 | 0.00 |
| ≥4 | 13 (41.9) | 13 (41.9) |  |  |
| Number of metastatic organs |  |  |  |  |
| ≤1 | 15 (48.4) | 20 (64.5) | 0.2003 | 0.33 |
| ≥2 | 16 (51.6) | 11 (35.5) |  |  |
| TPS |  |  |  |  |
| 1> | 3 (9.7) | 5 (16.1) | 0.4486 | 0.19 |
| 1< | 8 (25.8) | 6 (19.4) | 0.5435 | 0.15 |
| Unknown | 20 (64.5) | 20 (64.5) | 1.0 | 0.00 |
| Antacids |  |  |  |  |
| Yes | 30 (96.8) | 29 (93.5) | 0.9134 | 0.15 |
| No | 1 (3.2) | 2 (6.5) |  |  |
| Type of antibiotics |  |  |  |  |
| Beta-lactamase inhibitor | 8 (25.8) |  |  |  |
| Cephem | 10 (32.3) |  |  |  |
| Quinolone | 5 (16.1) |  |  |  |
| Penicillin | 5 (16.1) |  |  |  |
| Carbapenem | 1 (3.2) |  |  |  |
| ST | 1 (3.2) |  |  |  |
| Reason for antibiotics using |  |  |  |  |
| Infection | 12 (38.7) |  |  |  |
| Prophylactic using | 19 (61.3) |  |  |  |
| Abx, antibiotics; ECOG PS, European clinical oncology group performance status; ICI, immune checkpoint inhibitor; FP, 5FU plus cisplatin combination regimen; NLR, Neutrophil-lymphocyte ratio; TPS, tumor proportion score; ST, Trimethoprim-sulfamethoxazole | | | | |

Supplementary Table 5. ORR and DCR in propensity score matching

| Best response | Abx (+) (n = 28) | Abx (-) (n = 31) | *p*-value |  | irAE (-) (n = 33) | irAE (+) (n = 24) | *p*-value |
| --- | --- | --- | --- | --- | --- | --- | --- |
| CR | 0 | 0 |  |  | 0 | 0 |  |
| PR | 6 | 7 |  |  | 7 | 6 |  |
| SD | 6 | 13 |  |  | 8 | 10 |  |
| PD | 19 | 8 |  |  | 18 | 8 |  |
| ORR | 21.4 | 25.0 | 0.6014 |  | 21.2 | 25.0 | 0.7665 |
| DCR | 42.9 | 71.4 | 0.0118 |  | 45.5 | 66.7 | 0.0293 |
| Abx, antibiotics; irAE, immune related adverse event; CR, complete response; PR, partial response; SD, stable disease; PD, progressive disease; ORR, objective response rate; DCR, disease control rate | | | | | | | |

Supplementary Table 6. Immune related adverse events after propensity score matching

|  | Abx (+) (n = 31) | | Abx (-) (n = 31) | |  |
| --- | --- | --- | --- | --- | --- |
| irAE | Grade | | Grade | |  |
|  | Any grade (%) | Grade > 3 (%) | Any grade (%) | Grade > 3 (%) | *p*-value |
| Total | 8 (25.8) | 3 (9.7) | 16 (54.8) | 16 (54.8) | 0.037 |
| Interstitial pneumonia | 5 (16.1) | 3 (9.7) | 1 (3.2) | 1 (3.2) | 0.086 |
| Dermatitis | 1 (3.2) | 0 (0.0) | 4 (12.9) | 0 (0.0) | 0.162 |
| Hypothyroidism | 0 (0.0) | 0 (0.0) | 4 (12.9) | 0 (0.0) | 0.039 |
| Myositis | 1 (3.2) | 0 (0.0) | 0 (0.0) | 0 (0.0) | 0.313 |
| Arthritis | 1 (3.2) | 0 (0.0) | 1 (3.2) | 0 (0.0) | 1.0 |
| Colitis | 0 (0.0) | 0 (0.0) | 1 (3.2) | 1 (3.2) | 0.313 |
| Hypopituitarism | 0 (0.0) | 0 (0.0) | 4 (12.9) | 0 (0.0) | 0.039 |
| Hypoadrenalism | 0 (0.0) | 0 (0.0) | 1 (3.2) | 0 (0.0) | 0.313 |
| Type 1 diabetes mellitus | 0 (0.0) | 0 (0.0) | 1 (3.2) | 1 (3.2) | 0.313 |
| Abx, antibiotics; irAE, immune related adverse events | | | | | |
