# Supplementary material for: Association between antibiotic use, immune-related adverse events, and efficacy of immunotherapy in esophageal squamous cell carcinoma
Source: Int J Clin Oncol. 2026 Apr 27;31(7):1258–66. doi: 10.1007/s10147-026-03036-9 (PMC13303444; doi:10.1007/s10147-026-03036-9)
Supplement: Supplementary file 2 — Supplementary file2 Supplementary Fig. 1. Kaplan–Meier curves showing: a) PFS in all cases, b) PFS stratified by ICI treatment line, c) OS in all cases, and d) OS stratified by ICI treatment line. Supplementary Fig. 2. Cumulative incidence rate of irAEs in the antibiotics-treated and the antibiotics-untreated groups. This figure presents a time-to-event analysis evaluating the time to the first irAE occurrence on a per-patient basis. Accordingly, the number at risk differs from the total number of irAE events reported in Table 3, where all irAEs, including multiple events occurring in the same patient, were counted. Supplementary Fig. 3. Kaplan-Meier curves showing a) PFS and b) OS for the antibiotics-treated group and c) PFS and d) OS for the irAE-positive and irAE-negative groups after propensity score matching. (PPTX 153 KB) [file 10147_2026_3036_MOESM2_ESM.pptx]

## Slide 1
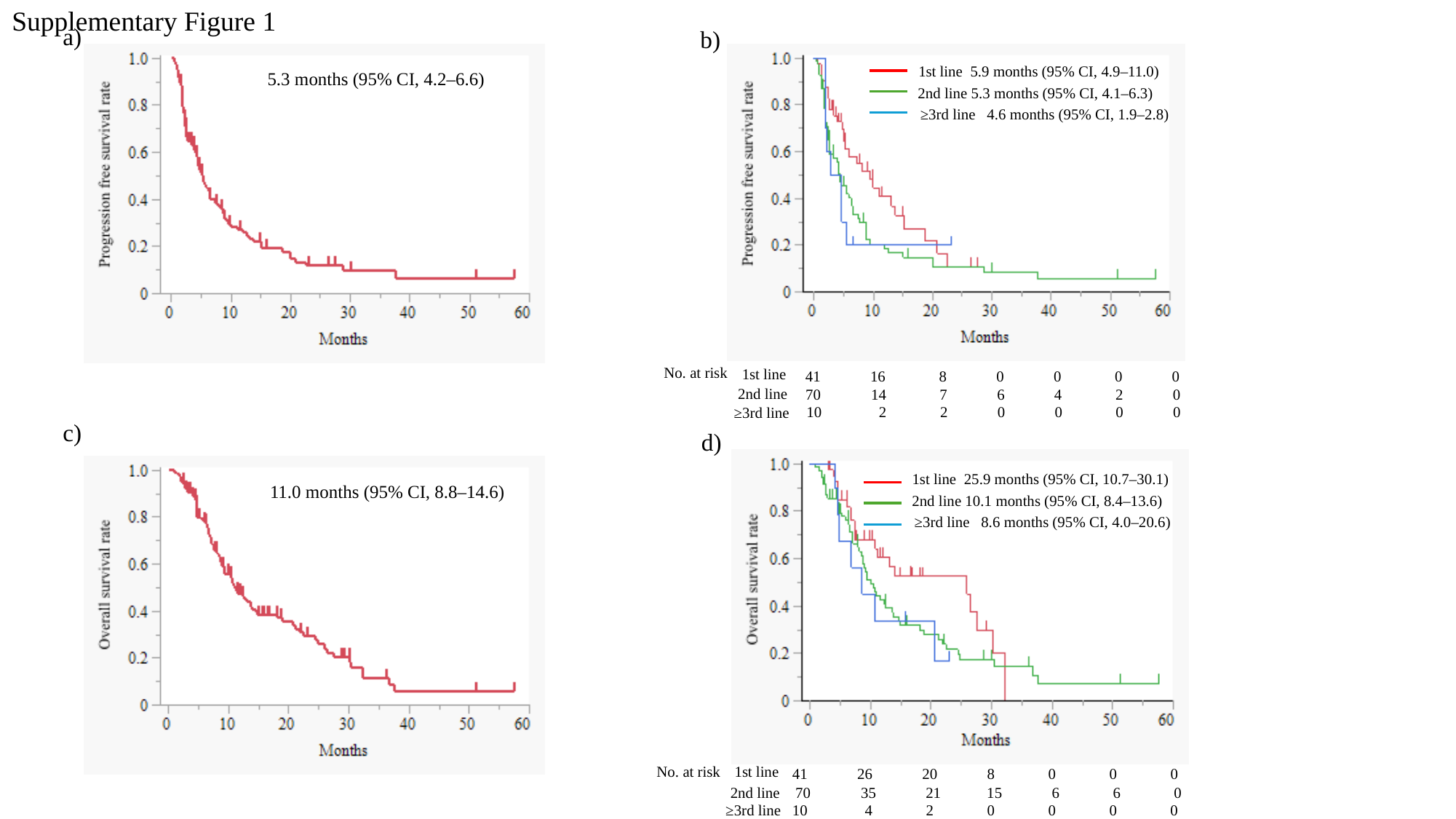

Supplementary Figure 1
a)
b)
1st line 5.9 months (95% CI, 4.9–11.0)
 5.3 months (95% CI, 4.2–6.6)
2nd line 5.3 months (95% CI, 4.1–6.3)
≥3rd line 4.6 months (95% CI, 1.9–2.8)
No. at risk
1st line
41 16 8 0 0 0 0
2nd line
 14 7 6 4 2 0
10 2 2 0 0 0 0
 ≥3rd line
c)
d)
1st line 25.9 months (95% CI, 10.7–30.1)
 11.0 months (95% CI, 8.8–14.6)
2nd line 10.1 months (95% CI, 8.4–13.6)
≥3rd line 8.6 months (95% CI, 4.0–20.6)
No. at risk
1st line
41 26 20 8 0 0 0
2nd line
 35 21 15 6 6 0
 ≥3rd line
10 4 2 0 0 0 0

## Slide 2
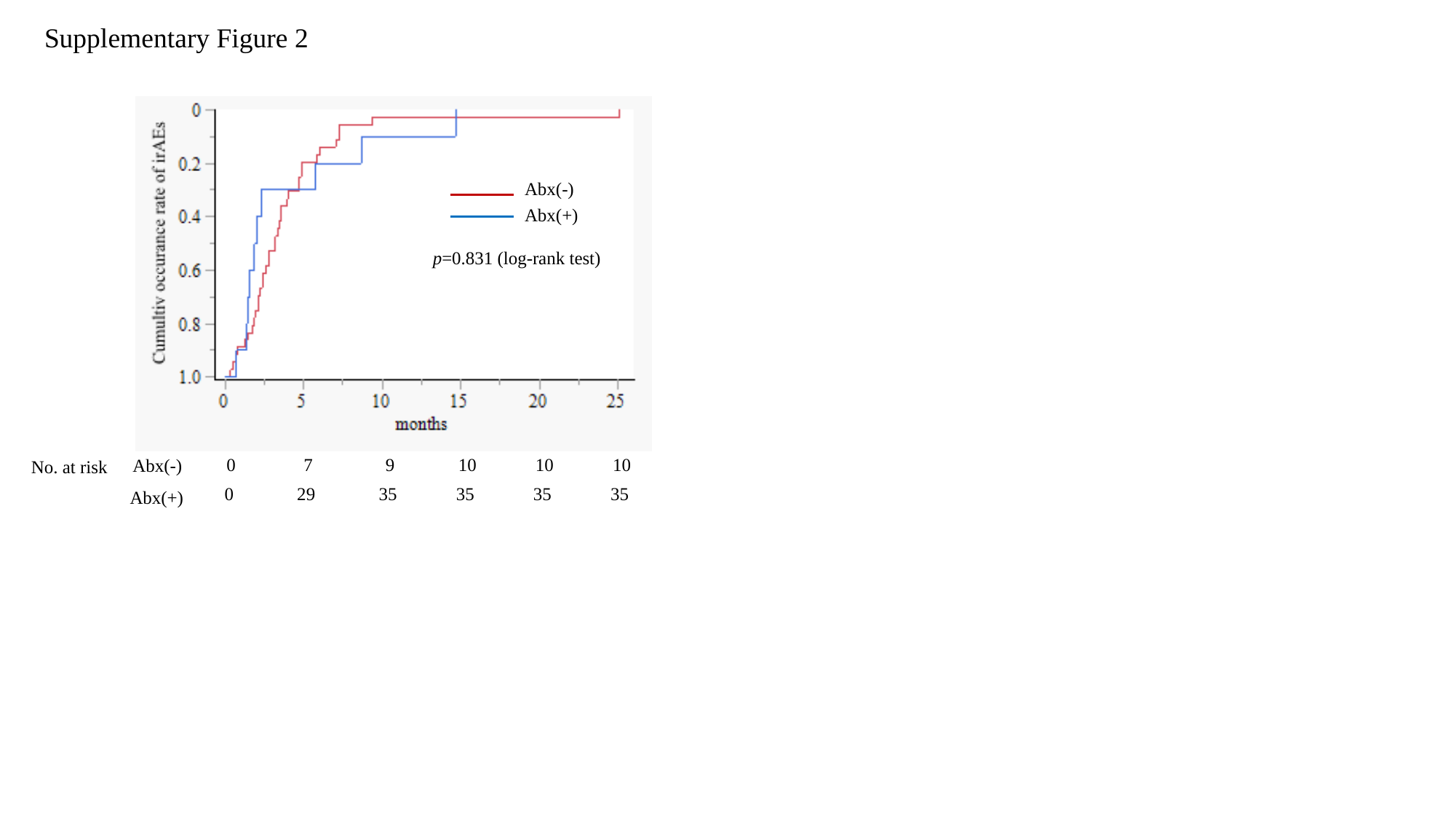

Supplementary Figure 2
Abx(-)
Abx(+)
p=0.831 (log-rank test)
0 7 9 10 10 10
Abx(-)
No. at risk
 0 29 35 35 35 35
Abx(+)

## Slide 3
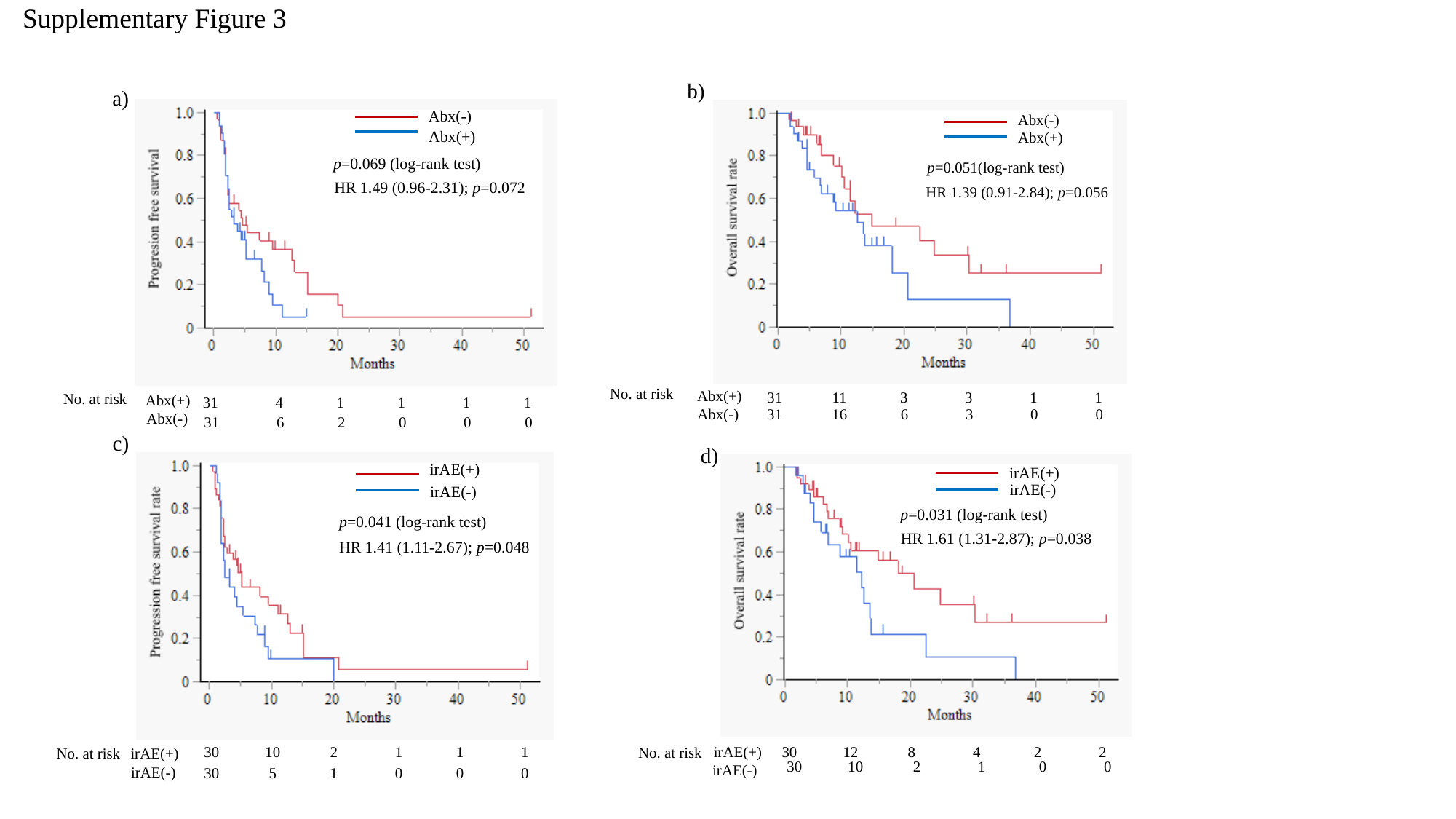

Supplementary Figure 3
b)
a)
Abx(-)
Abx(-)
Abx(+)
Abx(+)
p=0.069 (log-rank test)
p=0.051(log-rank test)
 HR 1.49 (0.96-2.31); p=0.072
 HR 1.39 (0.91-2.84); p=0.056
No. at risk
Abx(+)
31 11 3 3 1 1
No. at risk
Abx(+)
31 4 1 1 1 1
Abx(-)
31 16 6 3 0 0
Abx(-)
31 6 2 0 0 0
c)
d)
irAE(+)
irAE(+)
irAE(-)
irAE(-)
p=0.031 (log-rank test)
p=0.041 (log-rank test)
 HR 1.61 (1.31-2.87); p=0.038
 HR 1.41 (1.11-2.67); p=0.048
irAE(+)
30 12 8 4 2 2
30 10 2 1 1 1
No. at risk
No. at risk
irAE(+)
30 10 2 1 0 0
irAE(-)
irAE(-)
30 5 1 0 0 0
